# Protective effect of an improved immunization practice of mother-to-infant transmission of hepatitis B virus and risk factors associated with immunoprophylaxis failure

**DOI:** 10.1097/MD.0000000000004390

**Published:** 2016-08-26

**Authors:** Chong Wang, Chuan Wang, Zhi-Fang Jia, Xing Wu, Si-Min Wen, Fei Kong, Ke-Qin Hu, Jie Li, Jing Jiang, Jun-Qi Niu

**Affiliations:** a Department of Hepatology; b Division of Clinical Epidemiology, First Hospital of Jilin University, Changchun, Jilin, China; c Division of Gastroenterology and Hepatology, University of California, Irvine Medical Center, Orange, CA; d Department of Microbiology and Infectious Disease Center, School of Basic Medical Sciences, Peking University Health Science Center, Beijing, China; e Maternal and Child Health Center of Chaoyang District, Beijing, China.

**Keywords:** HBIG, hepatitis B virus, immunoprophylaxis, mother-to-infant transmission, vaccine

## Abstract

Although routine immunoprophylaxis has been known to reduce hepatitis B virus (HBV) transmission, immunoprophylaxis failure still occurs. The study aimed to investigate the protective efficacy of an improved immunoprophylaxis protocol to prevent mother-to-infant transmission of HBV and to explore the potential risk factors associated with immunoprophylaxis failure and low antibody response.

A prospective observational cohort study was conducted from July 2012 to April 2015. A total of 863 HBsAg-positive mothers and their 871 infants (8 pairs of twins) were included in the study. Two different hepatitis B vaccine doses (20 or 10 μg) were administered to the infants based on the hepatitis B e-antigen (HBeAg) status of their mothers. Simultaneously, hepatitis B immunoglobulin (HBIG) was administered to the infants. Initial injections of HBIG and the hepatitis vaccine were given within 2 hours after birth. Rates of HBV infections among the infants were evaluated at 7 months of age. Factors associated with immunoprophylaxis failure and low responses to vaccination were analyzed by unconditional logistic regression..

At 7 months of age, no immunoprophylaxis failure was observed in the 565 infants born to HBeAg-negative mothers. Among the 306 infants born to HBeAg-positive mothers, immunoprophylaxis failed in 16 infants (5.2%) of the infants and they were found to be HBsAg-positive. Further analysis showed that HBV DNA levels ≥10^8^ IU/mL [odds ratio (OR) = 4.53, 95% confidence interval (95% CI): 1.19–17.34], delayed vaccination (OR = 4.14, 95% CI: 1.00–17.18), and inadequate initial injections (OR = 7.69, 95% CI: 1.71–34.59) were independently associated with immunoprophylaxis failure. Adequate titers of antibody to HBsAg (anti-HBs, ≥100 mIU/mL) were present in 96.5% of immunoprophylaxis-successful infants. For full-term infants, birth weights <3000 g were correlated with low immune responses to vaccination.

This improved immunoprophylaxis protocol is effective in preventing perinatal transmission of HBV. Among infants with HBeAg-positive mothers, high HBV viral loads and inadequate and delayed initial injections were associated with immunoprophylaxis failure. The majority of the infants in our study produced adequate levels of protective anti-HBs titers after immunoprophylaxis. Additional efforts to further reduce perinatal transmission should be considered, especially for HBeAg-positive mothers.

## Abstract 2

Supplemental Digital Content is available in the text

## 1 Introduction

Hepatitis B virus (HBV) infection is a serious global health problem with approximately 240 million people persistently infected with HBV and 780,000 HBV-related deaths annually worldwide.^[1]^ The risk of becoming an HBV carrier is inversely related to the age of the infected patient. Chronic HBV infections persist in 1% to 5% of infected adults, 20% to 30% of young children, and up to 90% to 95% of perinatally infected infants. HBV infection early in life is likely to cause chronic disease and subsequent complications.^[2]^

A national survey conducted in China in 1992 showed that the prevalence of hepatitis B surface antigen (HBsAg) carriers was 9.67% among children <5 years old.^[3]^ Approximately 20 years ago, China implemented an HBV immunoprophylaxis strategy, which led to a 90% reduction (to 0.96%) in HBsAg carriers <5 years old by 2006.^[4,5]^ Approximately 90% of infants born to both HBsAg-positive and hepatitis B e-antigen (HBeAg)-positive mothers will become HBsAg carriers if no immunoprophylaxis is given.^[6]^ The most effective way to prevent mother-to-child transmission (MTCT) of HBV infection is by immunizing all susceptible individuals with adequate hepatitis B immune globulin (HBIG) and hepatitis B vaccines at birth, especially newborn infants born to HBV-positive mothers.^[2,7–9]^ Although administration of HBIG and the hepatitis B vaccine at birth has significantly reduced HBV infection rates, 0.75% to 9.66% of vertical HBV transmissions have not been eliminated by this combined intervention strategy.^[10–13]^ It has been suggested that providing antiviral therapy during the third trimester of pregnancy to mothers with high HBV viral loads would reduce the risk of perinatal transmission, but data regarding when and for how long this therapy should be administered are lacking.^[14]^

Although many studies on perinatal HBV prevention have been carried out using various prophylaxis protocols, the hepatitis B vaccine doses used in these studies have been ambiguous or inconsistent and ranged anywhere from 5 to 20 μg. Although high maternal HBV viral loads and HBeAg-positivity have been shown to be the predominant risk factors for immunoprophylaxis failure, only a few studies have evaluated the use of larger hepatitis B vaccine doses for infants born to HBeAg-positive mothers. Also, the initial injection time for HBIG and hepatitis B vaccine administration has not yet been strictly defined.^[15–18]^

In the present study, we conducted a prospective cohort of HBsAg-positive mothers and their infants to assess the protective efficacy of a modified immunoprophylaxis protocol. This modified protocol uses 2 different vaccine doses based on the maternal HBeAg status in combination with HBIG, and the injections are administered within 2 hours after birth. The HBV infection rate in infants and the potential risk factors associated with immunoprophylaxis failure and low protective antibody titers were evaluated.

## 2 Methods

### 2.1 Study design

This is a prospective, observational cohort study of HBsAg-positive mothers and their corresponding infants. On the basis of the status of HBeAg of mothers, different hepatitis B vaccine doses (20 or 10 μg) were given to the infants. Therefore, we limited our study to a nonrandomized, double-blind control design (with no appropriate control in the same HB vaccination group). For this reason, this study was not registered in the clinical trial registry. The study was approved by the Medical Ethics Committee of First Hospital of Jilin University (approval number 2012-098). Participants were informed of the purpose of the study and of their rights to keep information confidential. Written informed consent was obtained from each subject before participation. Patient records/information was anonymized and de-identified before analysis.

### 2.2 Study populations

For this study, all pregnant women were screened for HBV infection to improve the prevention and control of vertical HBV transmission. Pregnant women who tested positive for HBsAg at antenatal clinics in all of the districts and counties of Changchun were encouraged to go to the First Hospital of Jilin University for further HBV transmission prevention. Each pregnant woman was then given an immunization schedule that obstetricians and midwives at 25 cooperative birth hospitals would follow.

**Figure 1:**
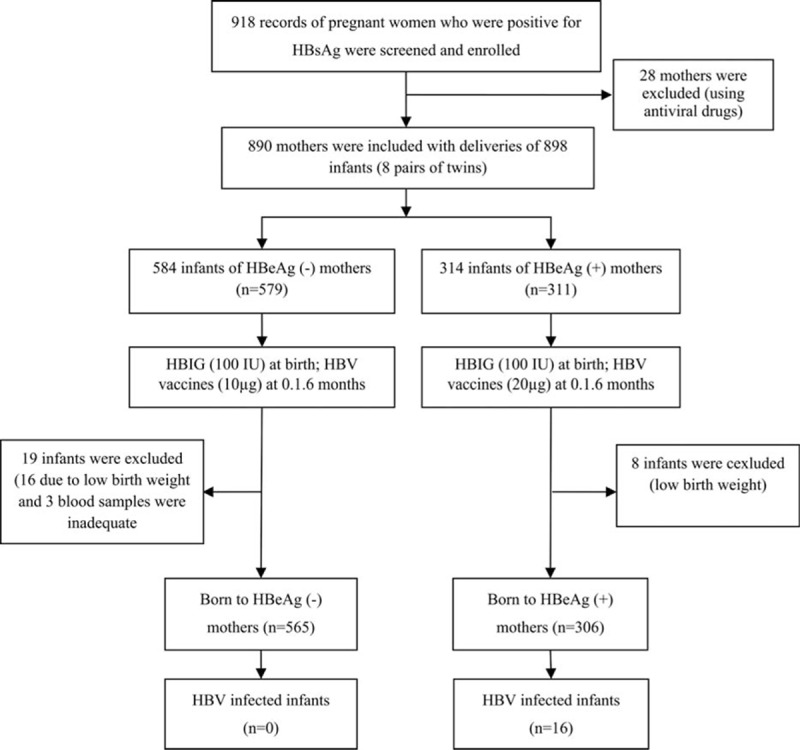
Flowchart depicting the study participants.

Pregnant women would not be included if they met any of the following exclusion criteria: having received antiviral medications or immune-modifying therapies during pregnancy; having coviral infections; and having any immunologically compromised conditions. Infant exclusion criteria were as follows: birth weight lower than 2500 g; and born before 37 weeks of gestational age. The study was carried out from July 2012 to April 2015, and infants were followed-up at 7 and 12 months of age. In total, 918 HBsAg-positive mothers registered and participated in the study, and 28 mothers and 27 infants were excluded due to the various reasons mentioned above. As a result, 871 infants were included in the final analysis. The flow chart depicting the study participants is summarized in Fig. 1.

### 2.3 Data collection

Two doctors were trained to complete the unified questionnaires when the mothers visit at the outpatient clinic. The questionnaires were administered face-to-face; the following sections were covered: socio-demographic data, gestational weeks, smoking and drinking status, family history of hepatitis B, antiviral therapy, and whether they were aware of HBV infection. Mothers were followed-up after delivery through telephone call, including delivery mode, feeding pattern, infant gender, infant weight, gestational age, and HBV vaccination dose with the time of injection. In addition, infants were continued 4 times of follow-up after birth (24 hours, 14 days, 1, and 6 months of age) for HBV vaccination information, and HBV serologic markers were measured at 7 and 12 months of age.

### 2.4 Immunization schedule

A combined passive and active immunization was administered to neonates born to HBsAg-positive mothers. Infants born to HBsAg-positive, HBeAg-negative mothers were injected with 100 IU of HBIG (Hualan Biological Engineering Inc., Xinxiang, China) and 10 μg of the hepatitis B vaccine (Hansenula polymorpha yeast-derived recombinant Hepatitis B vaccine; Dalian Hissen Bio-pharm Co., Dalian, China) within 2 hours after birth, followed by administration of 10 μg of the hepatitis B vaccine at 1 month and 6 months of age. Infants born to HBsAg-positive, HBeAg-positive mothers were injected with 100 IU of HBIG and 20 μg of the hepatitis B vaccine within 2 hours after birth, followed by administration of 20 μg of the hepatitis B vaccine at 1 month and 6 months of age. Administration of HBV vaccine and HBIG in accordance with the described protocol is defined as a timely and adequate injection.

### 2.5 Laboratory examinations

Venous blood samples were collected from pregnant women at approximately 1 month before delivery. Blood samples from infants were collected at the 7-month follow-up visit. All laboratory tests were performed in the central laboratory of the First Hospital of Jilin University. Serum samples from the mother and the infant were examined for 5 markers of HBV, including HBsAg, anti-HBs, HBeAg, anti-HBe, and anti-HBc, by chemiluminescent microparticle immunoassay (CMIA) with Abbott ARCHITECT i2000SR (Abbott Laboratories, North Chicago, IL). HBV DNA was assessed by the Roche Taqman HBV test (Roche Diagnostics, Grenzach, Germany) with a detection range from 8.9 to 109 IU/mL. Liver function tests were performed with a Synchron LXH20 autoanalyser (Beckman Coulter, Brea, CA).

### 2.6 Definition of immunoprophylaxis outcome

Seropositivity for HBsAg was used as the marker of HBV infection in infants. If an infant tested HBsAg-positive, the HBV DNA test was performed later to confirm immunoprophylaxis failure and HBV infection. The nonresponders, low responders, medium responders, and high responders were defined as HBsAg-negative infants with anti-HBs <10 mIU/mL, anti-HBs from 10 to 100 mIU/mL, anti-HBs from 100 to 1000 mIU/mL, and anti-HBs ≥1000 mIU/mL, respectively. We usually take anti-HBs ≥10 mIU/mL as a seroprotective response to vaccine. In this study, enough anti-HBs titer is still needed to prevent the babies from close contact infection with mothers, so anti-HBs titer ≥100 mIU/mL was considered a protective response, whereas a titer ≤100 mIU/mL was considered a low protective response.

### 2.7 Statistical analysis

A database of the mother-to-infant HBV transmission prevention cohort was established by the CapitalBio Life Science Data Management System (CB-LDMS-Sample). When the descriptive values were of normal distribution, *t* tests or nonparametric tests were used and data were expressed as mean ± standard deviation (SD). The Chi-square or Fisher exact test was used for categorical variables expressed in terms of n (%). Risk factors for immunoprophylaxis failure and low protective vaccine responses were analyzed by unconditional logistic regression with adjustment for possible confounders. The odds ratios (ORs) and 95% confidence intervals (CIs) were calculated to estimate risk. All statistical analyses were performed with the Statistical Package for Social Science (SPSS) for Windows version 18.0 (SPSS Inc., Chicago, IL). All *P* values were 2-tailed and a *P* value < 0.05 was considered statistically significant.

## 3 Results

### 3.1 Basic characteristics of mothers and infants

A total of 863 mothers and their corresponding 871 infants (8 pairs of twins) were included in the final analysis. Of these, 306 infants (35.1%) were born to HBsAg-positive, HBeAg-positive mothers, and 565 (64.9%) were born to HBsAg-positive, HBeAg-negative mothers. HBeAg-positive mothers were younger (27.71 ± 3.89 vs 28.98 ± 3.95 years old, *P* < 0.001) and had higher HBV DNA levels (7.75 ± 1.06 vs 2.71 ± 1.16 log_10_ IU/mL, *P* < 0.001), but had a lower rate of breastfeeding (30.1% vs 63.2%, *P* < 0.001) than HBeAg-negative mothers. Infants born to HBeAg-positive mothers and to HBeAg-negative mothers were similar in terms of gender distribution, birth weight, gestational age, and percentage of delayed vaccination. The basic characteristics of mothers and infants are summarized in Table 1.

**Table 1:**
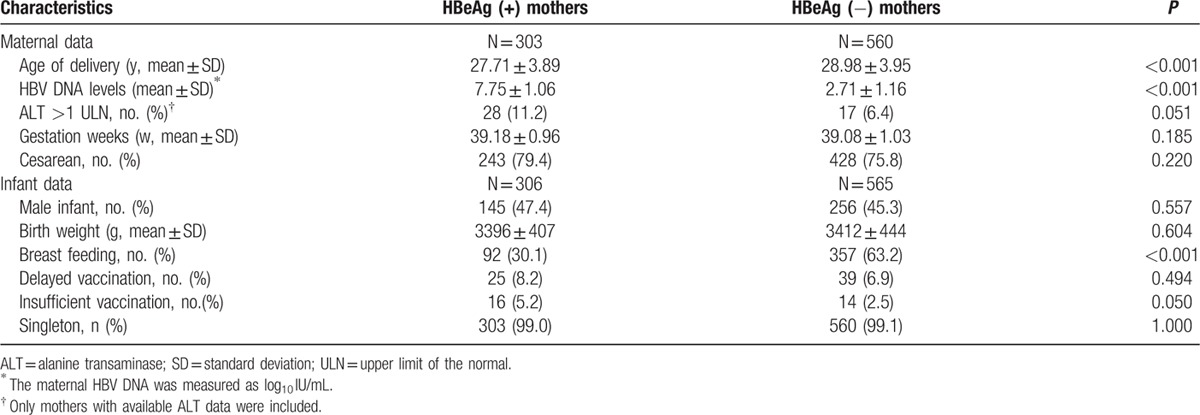
Basic characteristics of mothers and infants in the HBeAg-negative and HBeAg-positive groups.

### 3.2 The immunization outcome of the infants

Among 871 infants in this study, there were 16 infants who failed immunoprophylaxis and were HBsAg positive after the completion of hepatitis B vaccination schedule; the infant HBV infection rate was 1.84% (16/871). No immunoprophylaxis failure was observed in the 565 infants born to 560 HBeAg-negative mothers at 7 months. Among the 306 infants born to 303 HBeAg-positive mothers, the immunoprophylaxis failure (HBsAg-positive) rate was 5.2% (16/306). The HBV marker test results for infants at 7 months are summarized in Table 2. Four infants (3 in the 10 μg of HBV vaccine group and 1 in the 20 μg of HBV vaccine group) were HBeAg-positive at 7 months of age, but were HBeAg-negative at 12 months of age. All of the infants with immunoprophylaxis failure were born to HBeAg-positive mothers with HBV DNA >4 × 10^7^ IU/mL. Detailed information regarding the 16 infants with immunoprophylaxis failure and their mothers is summarized in Supplementary Table 1.

**Table 2:**
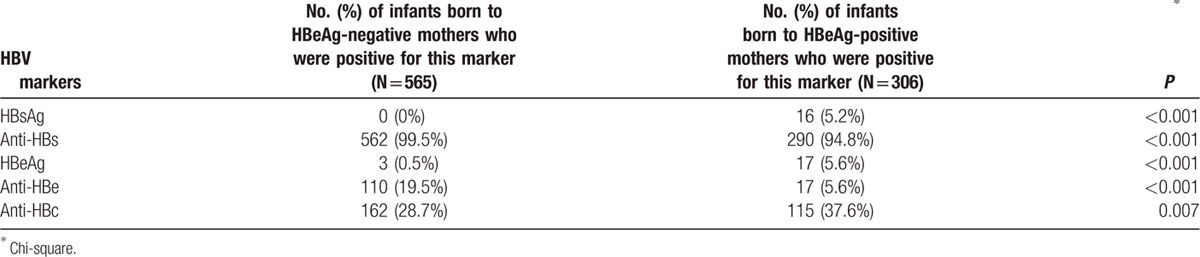
HBV marker test results for infants at 7 months of age.

### 3.3 Factors associated with immunoprophylaxis failure

As HBV transmission to infants born to HBeAg-negative mothers was all successfully prevented, we further analyzed factors related to immunoprophylaxis failure in infants born to HBeAg-positive mothers. The results of the univariate and multivariate logistic regression analyses are summarized in Table 3. After adjustment for other risk factors, multivariate regression analysis showed that mothers with higher HBV DNA levels (≥10^8^ IU/mL; Adjusted OR = 4.53, 95% CI: 1.19–17.34) and inadequate initial injections (Adjusted OR = 7.69, 95% CI: 1.71–34.59) were independently associated with an increased risk for immunoprophylaxis failure. Besides, nontimely initial injections (over than 2 hours) reached a borderline *significant* (Adjusted OR = 4.14, 95% CI: 1.00–17.18, *P* = 0.050). Other factors, including delivery mode, feeding pattern, infant gender, and birth weight, were not significantly associated with maternal HBV transmission.

**Table 3:**
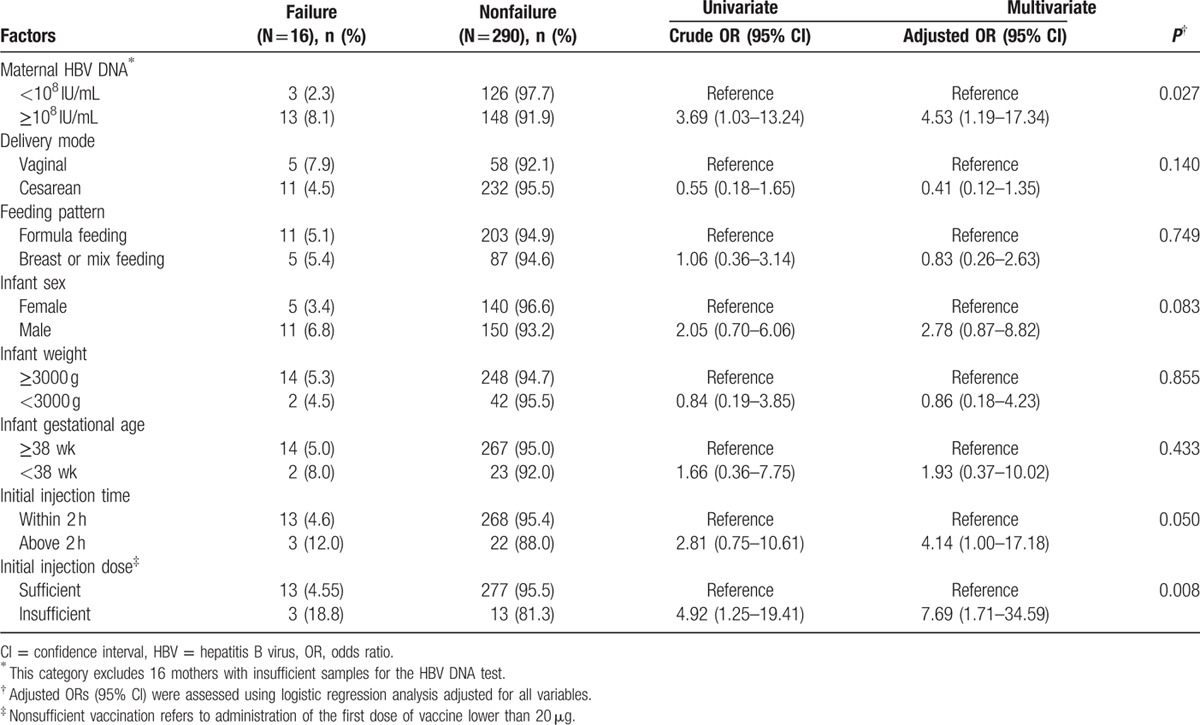
Factors influencing immunoprophylaxis failure in infants born to HBeAg-positive mothers (N = 306).

### 3.4 Factors correlated with the infant immune response to vaccination

We divided 855 HBV-negative infants into 2 groups. One group, the responder group, included 825 (96.5%) infants with anti-HBs titers ≥100 mIU/mL after completion of a series of 3 hepatitis B vaccine injections. The second group consisted of infants whose anti-HBs titers did not reach the protective level. Possible factors related to low immune responses to vaccination are summarized in Table 4. Infant birth weights <3000 g were correlated with low immune responses to vaccination (Adjusted OR = 2.47, 95% CI: 1.02–5.99; *P* = 0.045), whereas other factors such as the hepatitis B vaccine dose, maternal HBV DNA, delivery mode, feeding pattern, infant gender, gestational age, and initial injection time did not significantly correlate with a low response to vaccination. Besides, no difference was observed between different HepB injection dose and infants immune response distribution (Supplementary Table 2).

**Table 4:**
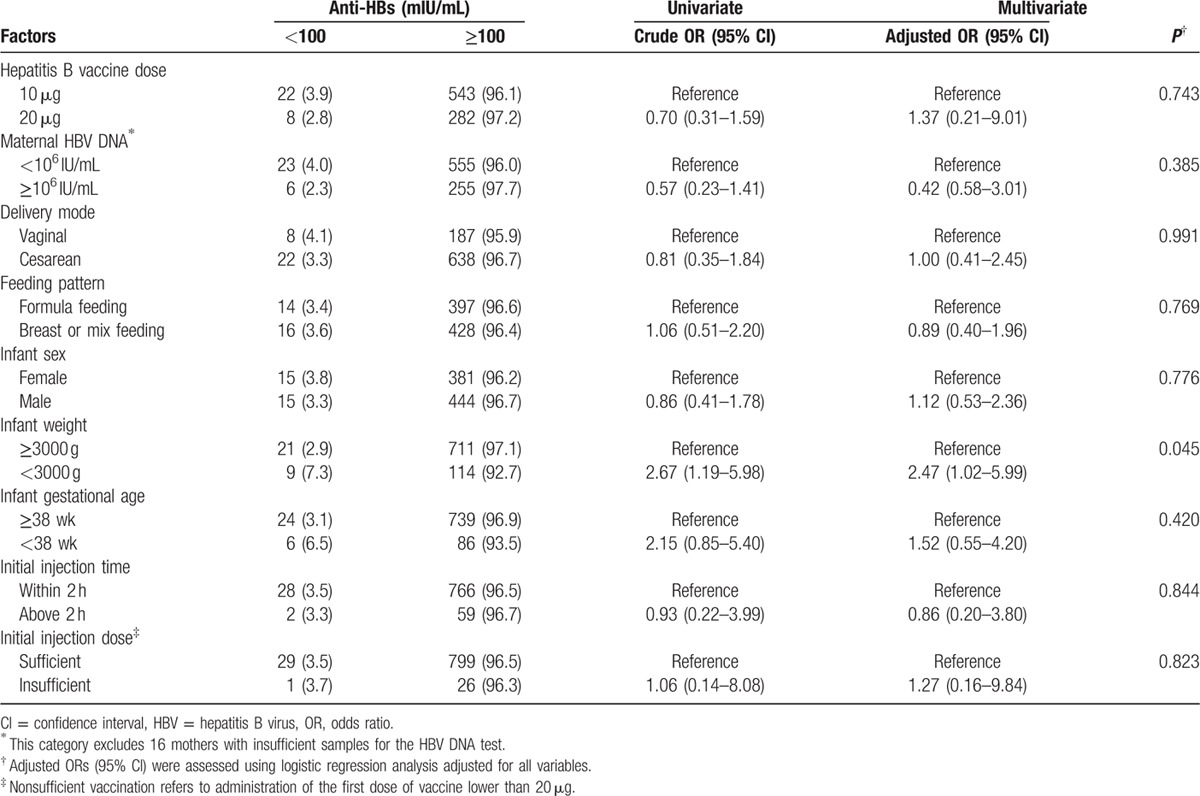
Possible factors related to low immune responses (anti-HBs ≤100 mIU/mL) to vaccination (N = 855).

### 3.5 Adverse events and safety

All of the infants who received the recombinant hepatitis B vaccine and HBIG were followed-up. No serious adverse events (SAEs) were reported, and only 36 (4.1%) of the newborns had temporary redness and fever following the first dose of the vaccine, but the symptoms resolved on their own without special treatment. No difference in the number of adverse reactions was observed between the 10 and 20 μg hepatitis B vaccine dose groups (4.4% vs 3.6%, *P* = 0.557).

## 4 Discussion

In this cohort study, infants born to HBsAg-positive mothers with different HBeAg statuses were given different immunization schedules, and the results were encouraging. None of the infants born to HBeAg-negative mothers were infected with HBV. All the immunoprophylaxis failure infants were born to mothers positive for both HBsAg and HBeAg, with a immunoprophylaxis failure rate of 5.2% observed. Maternal high HBV DNA levels and HB vaccine dose insufficiency along with delayed first injection were significantly associated with MTCT. Furthermore, our results showed that the majority of infants (96.5%) achieved protective level of antibodies at 7 months of age. Relatively low birth weight <3000 g in full-term babies was associated with weak response to vaccination.

Different from previous studies, we used 2 different doses of hepatitis B vaccine (20 and 10 μg) to the newborns based on their mothers’ HBeAg status instead of administered with 1 same dose of vaccine. Although an appropriate postnatal hepatitis B vaccine combined with HBIG was developed to prevent MTCT in 1992 in China, however, before the year of 2014, the HBV vaccination policy in Changchun city recommends that all newborn infants receive only 5 μg hepatitis B vaccine within 12 hours of birth and again at 1 and 6 months of age, and no additional prevention measures were implemented to mothers who were HBsAg carriers. This old strategy did not take the HBeAg status and HBV DNA level of the pregnant women into consideration. Given this background, it is very important to provide an improved immunoprophylaxis protocol to prevent MTCT of HBV. This benefit engineering was unique for that it provided an individual schedule to protect these in high-risk infants of HBV infection.

This study showed that immunoprophylaxis was successful for the majority of infants with only an overall MTCT rate of 1.84% (16/871), which was lower than previous reports with failure rates that ranged from 2.46% to 5.44%.^[18–20]^ Larger hepatitis B vaccine doses and timely vaccination as well as high cesarean rate might contribute to the low infection rate in our study. However, a higher immunoprophylaxis failure rate (5.2%) was observed in infants born to HBeAg-positive mothers. In a study by Kubo et al,^[9]^ among 624 infants born to 481 HBeAg-positive mothers, the overall infant HBV infection rate was 3.37%, which is lower than our results. Use of antiviral medications for parts of higher-risk populations in their study explains their low infection rates. In our study, to evaluate immunoprophylaxis efficacy only, we excluded all the mothers who had taken antiviral drugs during pregnancy.

The analysis of factors related to immunoprophylaxis failure in infants born to HBeAg-positive mothers revealed that high HBV DNA levels, insufficient hepatitis B vaccine doses, and delayed first injections were significantly associated with immunoprophylaxis failure. In our cohort, all the 16 blocking failure infants were born to HBeAg-positive mothers with HBV DNA >4 × 10^7^ IU/mL. After adjusting for other factors, HBV DNA was shown to be an independent risk factor for MTCT. This result is in accordance with previous data, which suggested that maternal HBV DNA >7–8 log_10_ copies/mL was significantly correlated with perinatal HBV transmission.^[17,21]^ Maternal HBV DNA >2 × 10^7^ IU/mL level had been recommended by AASLD (2009) and APASL (2012) for antiviral treatment during pregnancy.^[22,23]^ Our results showed that combined HBIG and hepatitis B vaccine immunoprophylaxis strategy is effective for HBeAg-negative mothers and for HBeAg-positive mothers with HBV DNA < 4 × 10^7^ IU/mL. Additional prophylaxis measures, such as late-pregnancy antiviral treatments, may be needed for HBeAg-positive mothers with high HBV viral load.

It was also found that vaccine doses and injection time are another 2 risk factors that are associated with maternal HBV transmission among infants born to HBeAg-positive mothers. The Chinese Society of Hepatology and Chinese Society of Infectious Diseases and Chinese Medical Association recommended that newborns of HBsAg positive mothers administering 10 μg recombinant yeast derived HB vaccine combined with HBIG 100 IU within 24 hours after birth (preferably within 12 hours), followed by 2 additional HB vaccine at 1 and 6 months, respectively.^[24]^ In most studies, a range of 5 to 20 μg of hepatitis B vaccine was given to newborns.^[17,25]^ In our study, the hepatitis B vaccine dose recommended for infants born to HBeAg-positive mothers was 20 μg, which was larger than the previous. Our results showed that this immunoprophylaxis was successful with a low infection rate and HB vaccination was well tolerated. However, immunoprophylaxis failure still occurred despite the use of combined HB vaccine and HBIG. Our data demonstrated that insufficient hepatitis B vaccine doses (<20 μg) were significantly associated with immunoprophylaxis failure (OR = 7.69, 95% CI: 1.71–34.59). Similarly, the Centers for Disease Control and Prevention (CDC) and the Advisory Committee on Immunization Practices (ACIP) also recommend that the HBV vaccine and HBIG be administered to at-risk infants within 12 hours of delivery.^[26]^ If their mothers are positive for both HBsAg and HBeAg, HBIG should be administered to newborns as soon as possible.^[27,28]^ However, there have been a variety of schedules for the initial passive–active immunization of the newborn, which vary anywhere from 3 to 24 hours after birth.^[15,17,29–32]^ Previous data from liver biopsies indicated that more than 2 hours is required for HBV to infect healthy liver cells^[33]^; therefore, the first injection of HBIG and the hepatitis B vaccine must be administered as early as possible after birth.^[11]^ In our study, the first dose of hepatitis vaccine and HBIG was required to be administered simultaneously within 2 hours after birth. The result showed that delayed initial injection of vaccine was correlated with immunization failure, which reach a borderline significant.^[34]^

HBeAg, anti-HBe, and anti-HBc pass through human placenta from mother to fetus and gradually disappear over time.^[35]^ In our study, 4 infants were HBeAg-positive at 7 months of age, but by 12 months of age, HBeAg was not detectable in these infants. The presence of anti-HBe before 12 months of age and the presence of anti-HBc before 24 months of age in infants may represent transplacental maternal antibodies rather than indicators of HBV infectious status.^[35]^ Therefore, HBV markers should not be evaluated in newborns immediately after birth, rather the HBV infection status of infants should be evaluated at 7 months of age and should continue thereafter to determine whether a chronic infection persists.

Because adequate primary vaccination provides long-term protection,^[36,37]^ it is necessary and important for infants to obtain adequate hepatitis B surface antibody titers at primary immunization to protect them from HBV infections and HBV-related diseases caused by potential future exposures. In this study, all the infants received the corresponding planned immunoprophylaxis protocol with the same brand of vaccine, the majority of the infants achieved a protective level, while still 3.5% of the infants became low immune responders. In this study, some factors that may be associated with low responsiveness to the HBV vaccine were analyzed, such as maternal HBV DNA, delivery mode, feeding pattern, infant gender, gestational age, birth weight, and vaccine dose. Besides, genetic factors such as the HLA group were also reported to be associated with non/low response to HB immunization.^[38,39]^ We found that only birth weights <3000 g was an independent predictive factor for weak response. Because the hepatitis B vaccine doses used in this study were greater than the doses used in most of the previous studies, and infants born prematurely weighing <2500 g may lead to incomplete immune fuction development,^[40]^ the vaccine-related safety may not be guaranteed; for this reason, only full-term infants with birth weights ≥2500 g were included in our study. The above results may be more significant if pre-term infants weighing <2500 g were also included. Therefore, it is essential to pay close attention to those low birth weight infants for the anti-HBs level, and hepatitis B booster vaccinations should also be considered when necessary.

This study is more convincing by the large sample size and accurate data collection. However, some limitations should be acknowledged. First, our observational cohort study was limited by the evaluation of the efficacy of immunization strategies; therefore, rigorous designed randomized controlled trials are needed in the future. Second, we can define the MTCT of our objects, but we have no evidence to confirm when the transmission actually occured (intrauterine infection, natal transmission, or postnatal transmission).

In conclusion, our study showed that this improved immunization is effective for preventing perinatal transmission of HBV, particularly for mothers who are HBeAg-negative. Maternal HBV DNA level and inadequate and delayed initial injections were the independent risk factors that predicted immunoprophylaxis failure. The majority of the infants in this study reached adequate protective antibody titers after immunoprophylaxis. A relatively low birth weight of <3000 g for full-term infants was associated with a weak response to vaccination. It is noteworthy that we are keeping on following up these children until 3 years old, and their long-term immunological outcome will be evaluated in the further research.

## Supplementary Material

Supplemental Digital Content

## References

[1] Goldstein ST Zhou F Hadler SC A mathematical model to estimate global hepatitis B disease burden and vaccination impact. *Int J Epidemiol* 2005; 34:1329–1339. 16249217 10.1093/ije/dyi206

[2] Beasley RP Rocks along the road to the control of HBV and HCC. *Ann Epidemiol* 2009; 19:231–234. 19344859 10.1016/j.annepidem.2009.01.017

[3] Liang XF Chen YS Wang XJ [A study on the sero-epidemiology of hepatitis B in Chinese population aged over 3-years old]. *Zhonghua Liu Xing Bing Xue Za Zhi* 2005; 26:655–658. 16471211

[4] Liang X Bi S Yang W Evaluation of the impact of hepatitis B vaccination among children born during 1992-2005 in China. *J Infect Dis* 2009; 200:39–47. 19469708 10.1086/599332

[5] Liang X Bi S Yang W Epidemiological serosurvey of hepatitis B in China: declining HBV prevalence due to hepatitis B vaccination. *Vaccine* 2009; 27:6550–6557. 19729084 10.1016/j.vaccine.2009.08.048

[6] Chang MH Natural history of hepatitis B virus infection in children. *J Gastroenterol Hepatol* 2000; 15 (Suppl):E16–E19. 10921376 10.1046/j.1440-1746.2000.02096.x

[7] Chen DS Toward elimination and eradication of hepatitis B. *J Gastroenterol Hepatol* 2010; 25:19–25. 20136972 10.1111/j.1440-1746.2009.06165.x

[8] Ni YH Chen DS Hepatitis B vaccination in children: the Taiwan experience. *Pathol Biol* 2010; 58:296–300. 20116181 10.1016/j.patbio.2009.11.002

[9] Kubo A Shlager L Marks AR Prevention of vertical transmission of hepatitis B: an observational study. *Ann Intern Med* 2014; 160:828–835. 24862434 10.7326/M13-2529 PMC4086733

[10] Lin X Guo Y Zhou A Immunoprophylaxis failure against vertical transmission of hepatitis B virus in the Chinese population: a hospital-based study and a meta-analysis. *Pediatr Infect Dis J* 2014; 33:897–903. 25361021 10.1097/INF.0000000000000315

[11] Kang W Ding Z Shen L Risk factors associated with immunoprophylaxis failure against mother to child transmission of hepatitis B virus and hepatitis B vaccination status in Yunnan province, China. *Vaccine* 2014; 32:3362–3366. 24793939 10.1016/j.vaccine.2014.04.045

[12] Tran TT Management of hepatitis B in pregnancy: weighing the options. *Cleve Clin J Med* 2009; 76 suppl 3:S25–S29. 19465706 10.3949/ccjm.76.s3.06

[13] Chen HL Lin LH Hu FC Effects of maternal screening and universal immunization to prevent mother-to-infant transmission of HBV. *Gastroenterology* 2012; 142:773–781. 22198276 10.1053/j.gastro.2011.12.035

[14] Lamberth JR Reddy SC Pan JJ Chronic hepatitis B infection in pregnancy. *World J Hepatol* 2015; 7:1233–1237. 26019737 10.4254/wjh.v7.i9.1233 PMC4438496

[15] Zou H Chen Y Duan Z Virologic factors associated with failure to passive-active immunoprophylaxis in infants born to HBsAg-positive mothers. *J Viral Hepat* 2012; 19:e18–e25. 22239517 10.1111/j.1365-2893.2011.01492.x

[16] Zou H Chen Y Duan Z Protective effect of hepatitis B vaccine combined with two-dose hepatitis B immunoglobulin on infants born to HBsAg-positive mothers. *PLoS One* 2011; 6:e26748. 22053208 10.1371/journal.pone.0026748 PMC3203892

[17] Wen WH Chang MH Zhao LL Mother-to-infant transmission of hepatitis B virus infection: significance of maternal viral load and strategies for intervention. *J Hepatol* 2013; 59:24–30. 23485519 10.1016/j.jhep.2013.02.015

[18] Alter MJ Epidemiology of hepatitis B in Europe and worldwide. *J Hepatol* 2003; 39 suppl 1:S64–S69. 14708680 10.1016/s0168-8278(03)00141-7

[19] Lavanchy D Hepatitis B virus epidemiology, disease burden, treatment, and current and emerging prevention and control measures. *J Viral Hepat* 2004; 11:97–107. 14996343 10.1046/j.1365-2893.2003.00487.x

[20] Chen DS Hepatitis B vaccination: the key towards elimination and eradication of hepatitis B. *J Hepatol* 2009; 50:805–816. 19231008 10.1016/j.jhep.2009.01.002

[21] Pan CQ Duan ZP Bhamidimarri KR An algorithm for risk assessment and intervention of mother to child transmission of hepatitis B virus. *Clin Gastroenterol Hepatol* 2012; 10:452–459. 22079509 10.1016/j.cgh.2011.10.041

[22] Lok AS McMahon BJ Chronic hepatitis B: update 2009. *Hepatology* 2009; 50:661–662. 19714720 10.1002/hep.23190

[23] Liaw YF Kao JH Piratvisuth T Asian-Pacific consensus statement on the management of chronic hepatitis B: a 2012 update. *Hepatol Int* 2012; 6:531–561. 26201469 10.1007/s12072-012-9365-4

[24] Chinese Society of H, Chinese Society of Infectious Diseases CMA. [The guideline of prevention and treatment for chronic hepatitis B (2010 version)]. *Zhonghua Gan Zang Bing Za Zhi* 2011; 19:13–24. 21272453 10.3760/cma.j.issn.1007-3418.2011.01.007

[25] Grosheide PM del Canho R Voogd M Anti-HBs levels in infants of hepatitis B carrier mothers after delayed active immunization with recombinant vaccine concomitant with DTP-polio vaccine: is there need for a second dose of HBIg? Dutch Study Group on Prevention of Neonatal Hepatitis B. *Vaccine* 1994; 12:1059–1063. 7998413 10.1016/0264-410x(94)90173-2

[26] Centers for Disease C, Prevention. Assessing completeness of perinatal hepatitis B virus infection reporting through comparison of immunization program and surveillance data—United States. *MMWR Morb Mortal Wkly Rep* 2011; 60:410–413. 21471948

[27] Publication WHO. Hepatitis B vaccines: WHO position paper—recommendations. *Vaccine* 2010; 28:589–590. 19896455 10.1016/j.vaccine.2009.10.110

[28] Mast EE Margolis HS Fiore AE A comprehensive immunization strategy to eliminate transmission of hepatitis B virus infection in the United States: recommendations of the Advisory Committee on Immunization Practices (ACIP) part 1: immunization of infants, children, and adolescents. *MMWR Morb Mortal Wkly Rep* 2005; 54 (RR-16):1–31. 16371945

[29] Danielsson N Fakakovikaetau T Szegedi E Improved immunization practices reduce childhood hepatitis B infection in Tonga. *Vaccine* 2009; 27:4462–4467. 19508908 10.1016/j.vaccine.2009.05.051

[30] Boot HJ Hahne S Cremer J Persistent and transient hepatitis B virus (HBV) infections in children born to HBV-infected mothers despite active and passive vaccination. *J Viral Hepatitis* 2010; 17:872–878. 10.1111/j.1365-2893.2009.01247.x 20051008

[31] Hu YW Liu HM Yuan J Effects of blocking intrauterine and perinatal hepatitis B virus infection with HBIG made at home or abroad. *Chinese J Pediatr* 2002; 40:733–735.

[32] Wang JS Zhu QR [Interruption of the transmission of hepatitis B virus from mother to babies]. *Zhonghua Gan Zang Bing Za Zhi* 2002; 10:308–310. 12223153

[33] Dai J Lu SC Yan LN [Investigation on the alteration of hepatitis B virus (HBV) markers in liver allograft of HBV related recipients in perioperative period]. *Zhonghua Gan Zang Bing Za Zhi* 2004; 12:331–333. 15225422

[34] Brown RS Jr McMahon BJ Lok AS Antiviral therapy in chronic hepatitis B viral infection during pregnancy: a systematic review and meta-analysis. *Hepatology* 2016; 63:319–333. 26565396 10.1002/hep.28302

[35] Wang JS Chen H Zhu QR Transformation of hepatitis B serologic markers in babies born to hepatitis B surface antigen positive mothers. *World J Gastroenterol* 2005; 11:3582–3585. 15962380 10.3748/wjg.v11.i23.3582 PMC4315966

[36] Madalinski K Kolakowska A Godzik P Current views on the persistence of immunity following hepatitis B vaccination. *Przegl Epidemiol* 2015; 69:47–51.147–150. 25862447

[37] Leuridan E Van Damme P Hepatitis B and the need for a booster dose. *Clin Infect Dis* 2011; 53:68–75. 21653306 10.1093/cid/cir270

[38] Li ZK Nie JJ Li J The effect of HLA on immunological response to hepatitis B vaccine in healthy people: a meta-analysis. *Vaccine* 2013; 31:4355–4361. 23887040 10.1016/j.vaccine.2013.06.108

[39] Yoon JH Shin S In J Association of HLA alleles with the responsiveness to hepatitis B virus vaccination in Korean infants. *Vaccine* 2014; 32:5638–5644. 25148772 10.1016/j.vaccine.2014.08.007

[40] Losonsky GA Wasserman SS Stephens I Hepatitis B vaccination of premature infants: a reassessment of current recommendations for delayed immunization. *Pediatrics* 1999; 103:E14. 9925860 10.1542/peds.103.2.e14

