# Supplementary material for: Protective effect of an improved immunization practice of mother-to-infant transmission of hepatitis B virus and risk factors associated with immunoprophylaxis failure
Source: Medicine (Baltimore). 2016 Aug 26;95(34):e4390. doi: 10.1097/MD.0000000000004390 (PMC5400313; doi:10.1097/MD.0000000000004390)
Supplement: Supplemental Digital Content [file medi-95-e4390-s001.doc]

**Supplemental Digital Content**

Supplemental Digital Content 1. Table. Characteristics of HBV-infected infants and corresponding mothers. Supplemental Digital Content 2. Table. Distribution of different responders in mothers with different status.

**Supplementary Table 1.** Characteristics of HBV-infected infants and corresponding mothers.

| No | Sex* | Birth type | Vaccine dose (µg) | Initial injection time | Birth Weight (kg) | Feeding practice† | Age at delivery (yr) | Viral load (log_10_ IU/ml) | Threatened abortion or preterm labor |
| --- | --- | --- | --- | --- | --- | --- | --- | --- | --- |
| 1 | M | Cesarean | 20 | < 2 h | 3.2 | FF | 27 | 8.5 | no |
| 2 | M | Cesarean | 10 | < 2 h | 2.8 | FF | 33 | 7.9 | no |
| 3 | M | Cesarean | 20 | < 2 h | 3.6 | BF | 25 | 8.3 | no |
| 4 | M | Cesarean | 20 | < 2 h | 4.0 | FF | 33 | 8.2 | no |
| 5 | F | Vaginal | 10 | < 2 h | 3.3 | FF | 24 | 8.2 | no |
| 6 | M | Cesarean | 20 | 12-24 h | 3.8 | FF | 33 | 8.5 | no |
| 7 | F | Vaginal | 20 | < 2 h | 3.9 | BF | 28 | 8.0 | no |
| 8 | F | Cesarean | 10 | > 24 h | 3.5 | FF | 26 | 7.8 | no |
| 9 | M | Vaginal | 20 | < 2 h | 3.4 | FF | 24 | 7.6 | no |
| 10 | M | Vaginal | 20 | < 2 h | 2.6 | FF | 30 | 8.4 | no |
| 11 | M | Cesarean | 20 | 12-24 h | 3.6 | BF | 27 | 8.5 | no |
| 12 | M | Cesarean | 20 | < 2 h | 4.1 | BF | 24 | 8.7 | no |
| 13 | M | Cesarean | 20 | < 2 h | 3.8 | FF | 28 | 8.0 | no |
| 14 | M | Cesarean | 20 | < 2 h | 3.0 | FF | 31 | 8.5 | no |
| 15 | F | Cesarean | 20 | < 2 h | 3.2 | FF | 34 | 8.5 | no |
| 16 | F | Vaginal | 20 | < 2 h | 3.5 | BF | 27 | 8.4 | no |

* M, male; F, female.

† BF, breast feeding; FF, formula feeding; Mixed feeding was [classified](http://www.iciba.com/classified) to BF.

**Supplementary Table 2. Distribution of different responders in mothers with different status.**

| HepB injection dose | Number (%) of infants | | | | χ² | *P* value |
| --- | --- | --- | --- | --- | --- | --- |
|  | non-responder | low- responder | medium-responder | high- responder |  |  |
| 10 ug | 2 (0.4) | 20 (3.5) | 177 (31.3) | 366 (64.8) | 1.799 | 0.726 |
| 20 ug | 0 (0) | 8 (2.8) | 98 (33.8) | 184 (63.4) |  |  |
